# The comparison of the Effect of double reverse traction repositor (DRTR) and traction table assisted Anterograde Intramedullary nail in treatment of femoral shaft fractures

**DOI:** 10.1186/s12891-023-06421-x

**Published:** 2023-04-18

**Authors:** Wei Song, Yueying Wang, Weihao Chen, Zhenqian Zhang, Xuzhou Liu, Guoji Ou, Benqiang Cheng, Hongsheng Lin

**Affiliations:** 1 Department of Orthopaedics, The First Affiliated Hospital of Jinan University, Guangzhou, China; 2 Department of Orthopaedics, The First people’s Hospital of Zhaoqing, Zhaoqing, China; 3 Clinical Laboratory of the First People’s Hospital of Zhaoqing, Zhaoqing, China

**Keywords:** Femoral shaft fractures, Anterograde Intramedullary nail, Double reverse traction repositor, Traction table, Surgical technique

## Abstract

**Objective:**

The objective of this study was to compare the clinical efficacy of DRTR (Double Reverse Traction Repositor, DRTR)and traction table in the treatment of femoral shaft fractures with the aid of AN-IMN (Antegrade intramedullary nailing).

**Patients and Methods:**

In this study, patients with femoral shaft fractures admitted to the Department of Orthopedics at Zhaoqing First People’s Hospital from May 2018 to October 2022 were recruited. All patients were treated with anterograde intramedullary nailing, with 23 patients in the DRTR-assisted group and 21 patients in the traction table-assisted group. The demographic characteristics, fracture classification, intraoperative data, postoperative data, and prognostic indicators of the two groups were recorded and analyzed retrospectively. All procedures were performed by the same team of experienced physicians.

**Results:**

All the patients in the two groups were followed up for more than 12 months. Both traction methods could provide stable traction for the operator during AN-IMN, and there was no significant difference in demographic characteristics and fracture classification. The intraoperative fluoroscopy times and opening reduction rate of the DRTR group were lower than those of the traction table group (P < 0.05), and the postoperative Harris Hip Score, as well as the Lyshol Lysholm knee function Score of the DRTR group, were significantly higher than the traction table group members (P < 0.05). Postoperative complications such as perineal soft tissue injury and lateral femoral cutaneous nerve injury occurred in the traction table group, but not in the DRTR group.

**Conclusion:**

DRTR can safely and effectively provide continuous and stable traction in the femoral shaft fractures surgery, and outperforms the traction table in the number of intraoperative fluoroscopy, opening reduction rate, reduction of complications, and postoperative joint function score.

## Introduction

The incidence of infra-isthmal femoral shaft fractures is high in adult limb fractures, accounting for 4.6%, of femoral shaft fractures, or one-fifth of the total [2]. The femoral shaft fracture refers to the area from the lower edge of the transverse line of the lesser trochanter to the upper edge of the line of the medial and lateral epicondyle of the femur, while the infra-isthmal femoral shaft extends from the pulp cavities Isthmus to the upper edge of the line of the medial and lateral epicondyle of the femur [1]. Currently, surgical treatment is the preferred approach for infra-isthmal femoral shaft fractures, including plate fixation, external fixation, intramedullary nail, etc.

The intramedullary nail device has been documented to have been first used by the ancient Egyptians to treat adult fractures [2]. With the development of trauma orthopedics, the advantages of intramedullary nailing have been increasingly recognized, such as a smaller surgical scar, lower infection rate, earlier ambulation of patients, less blood loss, less interference with the fracture site and surrounding soft tissue, and lower fracture nonunion rate, making it as the *Gold Standard* for the treatment of long bone fractures [3]. At present, two types of intramedullary nailing are widely used in clinical practice: retrograde intramedullary nailing (RE-IMN) and Antegrade intramedullary nailing (AN-IMN). RE-IMN is usually preferred for the treatment of early-stage infra-isthmal femoral shaft fractures due to its higher satisfaction rates. However, with an increasing number of cases, certain complications have been observed, such as articular cartilage damage during nail placement, resulting in chronic knee pain and knee infection, which can be effectively addressed by AN-IMN [4].

Maintaining the stability of axial traction of the femoral shaft during intramedullary nail insertion is crucial for fracture reduction. Traditionally, traction tables have been used to provide traction during intramedullary nail surgery for femoral shaft fractures through the confrontation between the foot and the perineal column [5]. However, they can produce potential complications such as perineal nerve damage, foot traction injury, and crush syndrome. To address this, our hospital has introduced the double reverse traction frame, and a comparison of the clinical efficacy of AN-IMN surgery assisted by double reverse traction frame and traction table in treating femoral shaft fractures has been made.

Zhang [6] designed the DRTR to assist in the reduction and maintenance of femoral fractures. Currently, it is extensively used in the surgical treatment of long bone diaphysial fractures by orthopedic surgeons. However, the efficacy of DRTR and orthopedic traction table in AN-IMN is yet to be fully elucidated. Therefore, this study aims to compare the clinical efficacy of DRTR and traction table in the treatment of infra-isthmal femoral shaft fractures with AN-IMN.

## Patients and methods

Patients with femoral shaft fractures who were hospitalized in the Department of Trauma and Orthopedics of the First People’s Hospital of Zhaoqing between May 2018 and May 2022 were recruited for this study. All patients were treated with AN-IMN surgery assisted by DRTR or an orthopedic traction bed.

The inclusion criteria for sampling were:

1. Aged between 18 and 80 years;
2. Unilateral middle and lower femoral shaft fracture;
3. The follow-up time of more than 12 months;

The exclusion criteria were:

1. Pathological fractures;
2. Open fractures;
3. Combined with fracture complications, such as vascular and nerve injury;
4. Old femoral shaft fractures;
5. Patients who refused to use the traction table or DRTR;
6. Patients with incomplete data.

After review, 44 patients meeting the criteria were randomly divided into two groups. The first group underwent AN-IMN surgery assisted with DRTR, while the second group underwent AN-IMN surgery with the aid of a traction table. The study was approved by the Institutional Review Board of the First People’s Hospital of Zhaoqing and all participants provided written consent. This study was conducted in accordance with the ethical standards and the Declaration of Helsinki.

A total of 44 patients (34 men and 10 women aged from 18 to 80 years, mean 37.6 ± 17.8 years) were enrolled in the study following inclusion and exclusion criteria. Of these, 28 had OA/OTA classification Type 32-A type and 16 had Type 32-B. After hospital admission, the full-length anteroposterior and lateral radiographs of the femur were improved, and the preoperative evaluation was prepared.

### Surgical Techniques and Follow-Up protocol

The surgery was performed by the same physicians with more than 7 years of experience in traumatic orthopedic surgery.

#### Traction table group

**Fig. 1:**
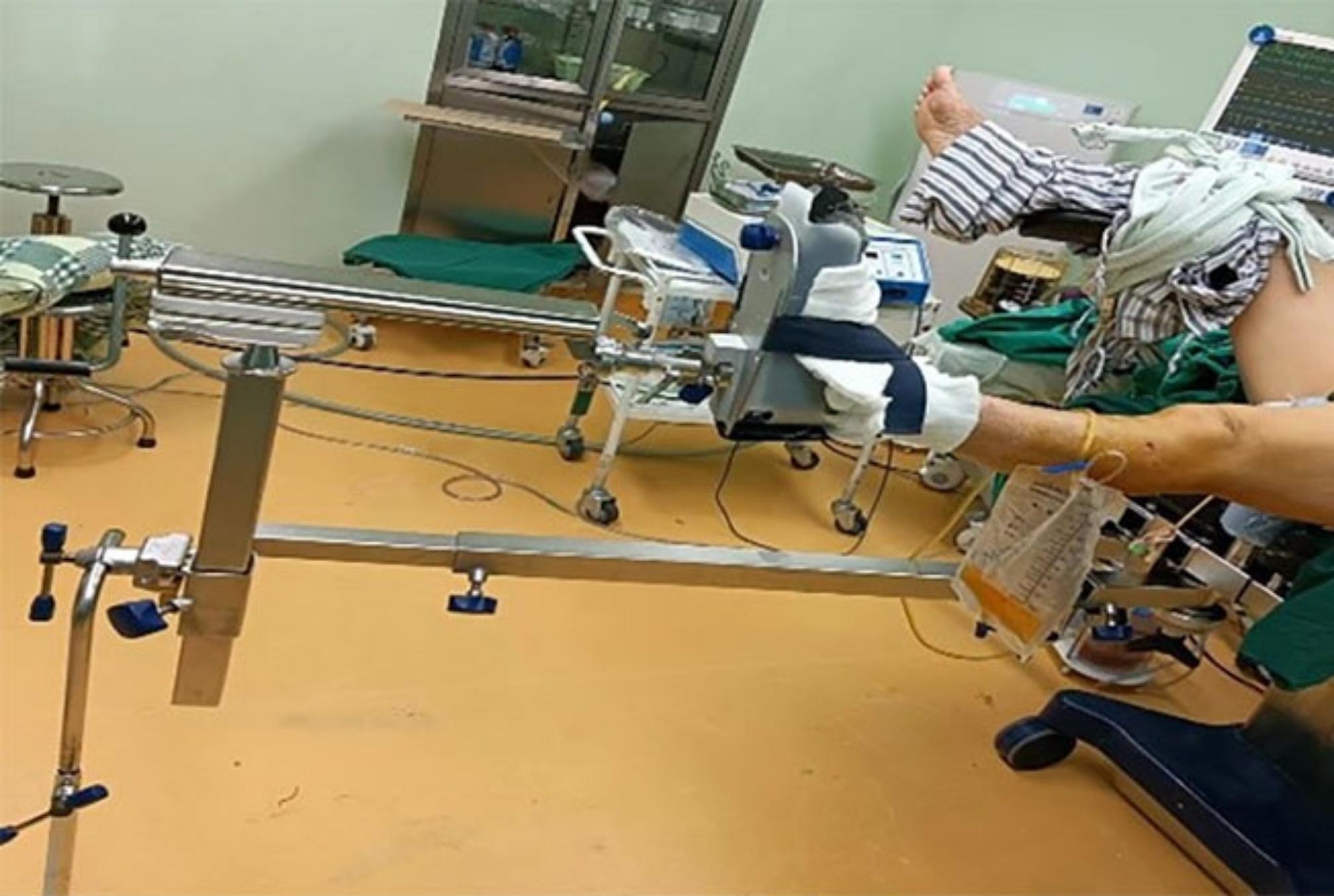
Application of orthopedic traction table

After anesthesia, the patient was placed on the traction table bed (Fig. 1), with the affected lower limb placed in the traction frame for intraoperative position change and continuous traction and the healthy limb extended on the abductor frame for C-arm fluoroscopy of the femur side. The perineum and foot of the affected limb serve as two fulcrums for the femur to gain continuous traction.

The traction force, rotation, adduction, and abduction angles of the affected limb were then adjusted by the rotating rod of the foot. Once the fracture was reduced by C-arm fluoroscopy, the disinfected tissue was tiled, a longitudinal incision was made 5 cm above the greater trochanter and the guide needle was inserted into the marrow cavity from the vertex. The pulp cavity reaming was carried out, and the intramedullary nail of appropriate size and length was placed in the femoral bone marrow cavity after confirming the guide needle’s entry into the medullary cavity by C-arm fluoroscopy. If the guide wire was unable to pass through the distal medullary cavity, due to poor reduction or block of bone fragments, open replacement would be necessary. Finally, proximal and distal locking screws were inserted. C-arm was often used to assist in the placement of the distal locking nail due to the bending-forward phenomenon of adult femurs, which made it difficult for the preinstalled catch bar to accurately locate the intramedullary nail in the medullary cavity.

**Fig. 2:**
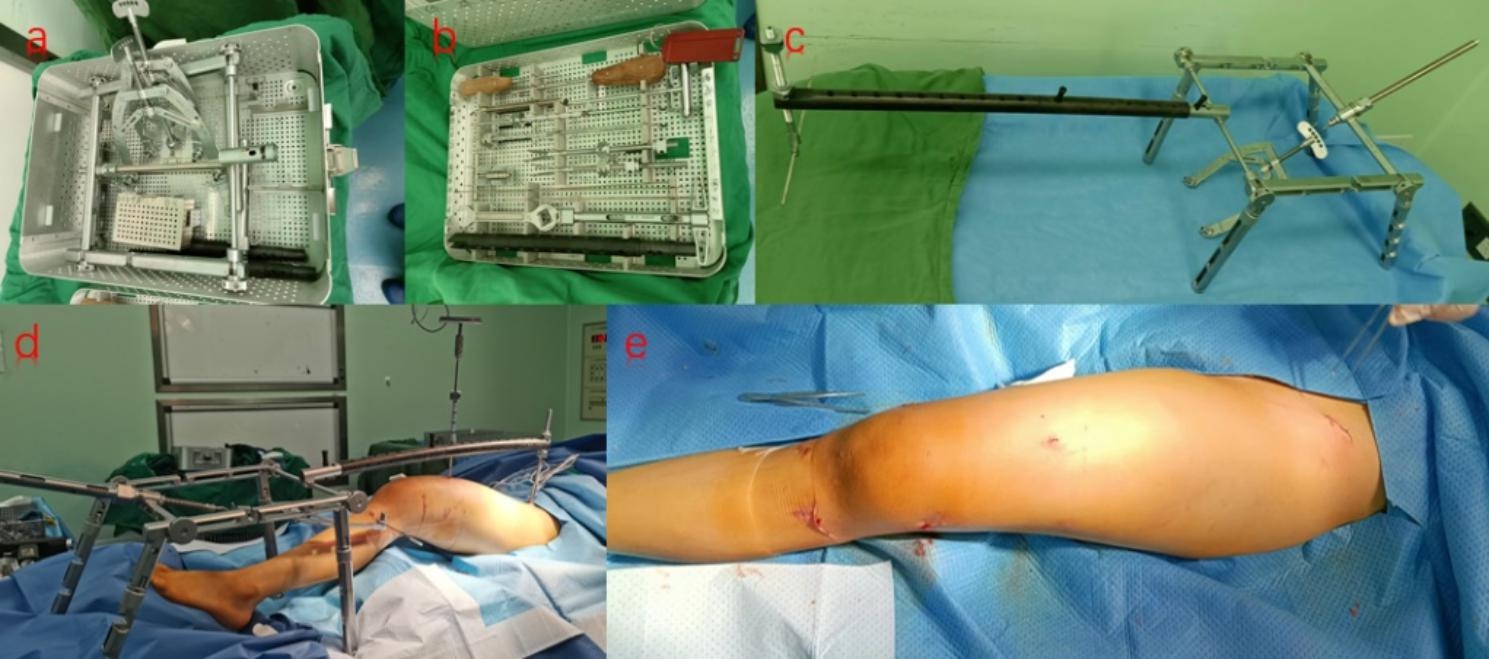
Application of DRTR: (**a**, **b**) Composition and assembled shape of DRTR; (**c**) The general view post DRTR installation; (**d**) Intraoperative closed reduction of the fracture through the reduction device; (**e**) Postoperative wound condition of the patient

The DRTR (Fig. 2) which mainly consists of a proximal fixator, a connecting rod, a bracket, a traction bow, a traction needle, and a proximal connecting device was used with the patient lying on an extended operating table. After tiling the anesthesia cloth, the anterior superior iliac spine was identified and a 3 cm oblique incision was made to expose the bone surfaces of both sides of the iliac crest. The proximal fixator of the DRTR was then fixed at the anterior superior iliac spine through transverse screws, which had to pass through the cortical bones on both sides of the anterior superior iliac spine to prevent avulsion fractures during traction. To ensure the intramedullary nail placement, the fixation point of the distal fixator was usually made 2 cm below the tibial tuberum, and a 2.5 mm diameter Kirschner wire was screwed using an electric drill and attached to the traction bow.

**Fig. 3:**
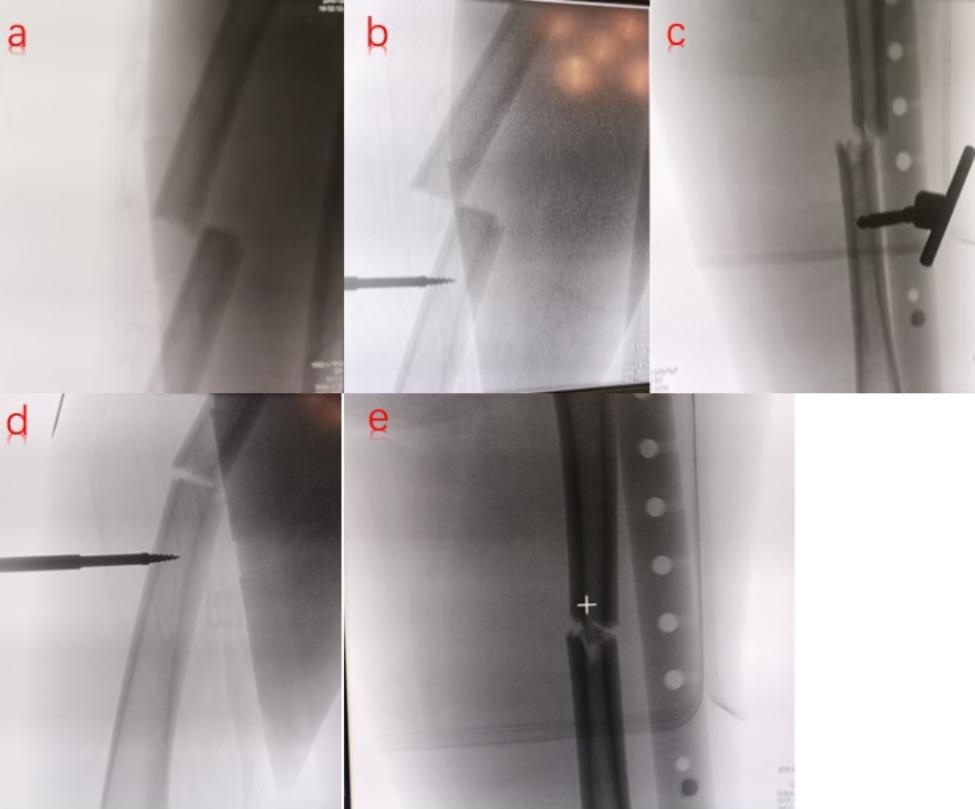
Reduction of fractures in the middle and lower femoral shaft by DRTR: (**a**) Lateral radiographs of the fracture before reduction; (**b**) Lateral radiographs of the distal femoral fracture inserted with a Kirschner steel needle; (**c**) Anterior radiographs of the distal femur fracture inserted with a Kirschner steel needle; (**d**) Lateral radiographs of the femur after reduction; (**e**) Anteroposterior radiograph after reduction

Finally, the proximal and distal fixators were assembled by connecting rods (Fig. 3). After assembly, the proximal traction site of DRTR was at the ilium, and the distal traction site was at the tibial tubercle. Then, the handle of the distal DRTR was rotated until the quadriceps femoris was maximally strained, thereby generating the maximum traction force. Under this traction force, most of the rotational displacement could be improved, and the overlapping displacement could be corrected by raising the affected limb. Lateral displacement could be corrected using manipulative reduction techniques by the operator.

After successful reduction, an intramedullary nail of appropriate length and diameter was placed following the same process as the traction table. The proximal and distal locking devices were then installed and the proximal screw and the tail nail were placed after DRTR had been removed. Finally, the layered incisions were sutured once the position of the implant and the fracture reduction had been confirmed via the C-arm.

### Follow-up protocol

Antibiotics were used within 24 h to prevent wound infection. Rivaroxaban was given orally as one tablet (10 mg) daily to prevent thrombosis during perioperative period. What’s more, during the pre-operation, the patient wore elastic stockings on the lower leg and performed ankle pump exercises. And during the post operation, the patients were encouraged to perform isometric contraction of quadriceps femoris and ankle pump exercise independently. CPM was also performed to assist the patients to perform functional exercise of the affected limb. And The follow-up period was at least 12 months.

**Fig. 4:**
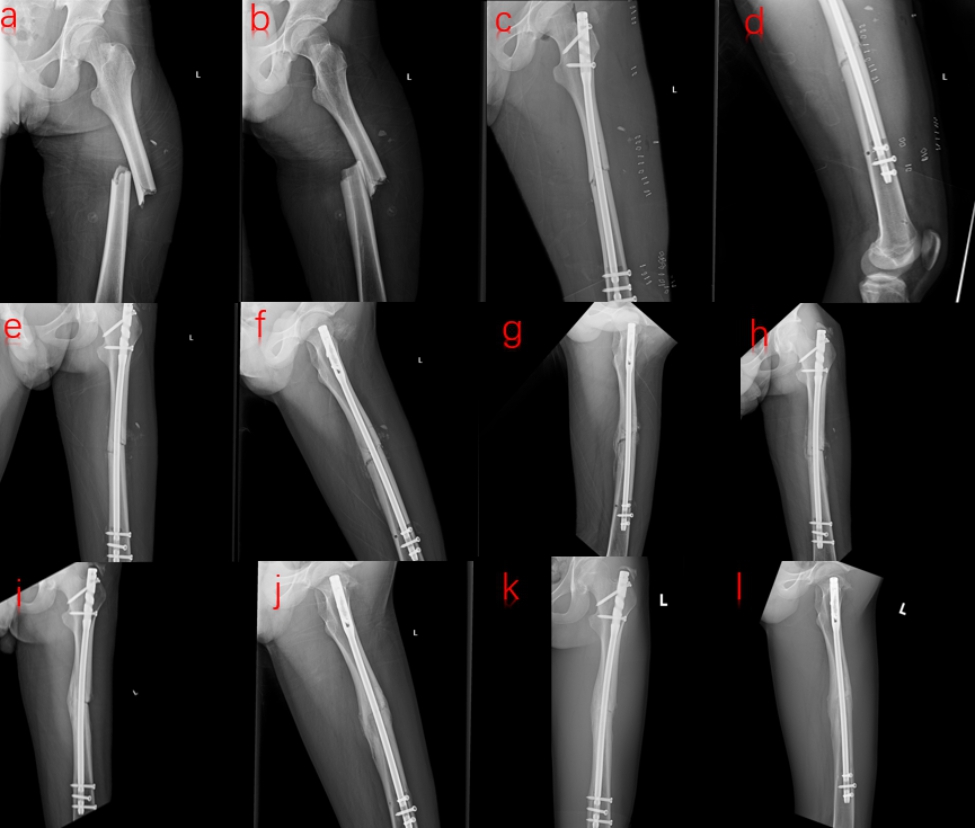
Radiographs of a case after DRTR: (**a**, **b**) Preoperative anterior-posterior (AP) and lateral views; (**c**, **d**) AP and lateral views on the second postoperative day; (**e**, **f**) AP and lateral views one month postoperatively; (**g**, **h**) AP and lateral views three months postoperatively; (**i**, **j**) AP and lateral views 6 months postoperatively; (**k**, **l**) AP and lateral views 12 months postoperatively

**Fig. 5:**
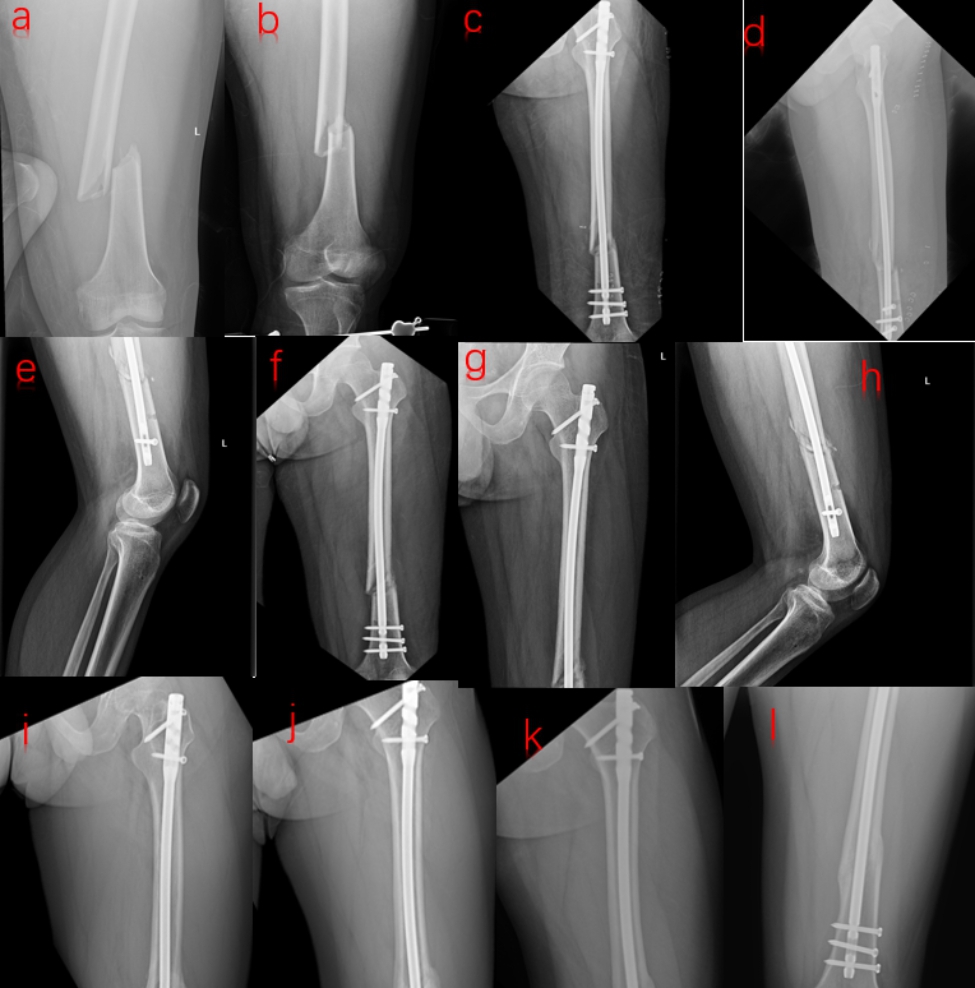
Radiographs of a case following traction table group: (**a**, **b**) The preoperative anterior-posterior (AP) and lateral views; (**c**, **d**) AP and lateral views on the second postoperative day; (**e**, **f**) AP and lateral views one month postoperatively; (**g**, **h**) AP and lateral views three months postoperatively; (**i**, **j**) AP and lateral views six months postoperatively; (**k**, **l**) AP and lateral views 12 months postoperatively

Properly and partially weight bearing exercises were recommended during the early stages of recovery, with a gradual increase in load until the fracture line disappeared. After six months of fracture healing, the follow-up frequency would be reduced. It was defined as fracture nonunion if there was still a significant fracture line 9 months after surgery and no progress was perceived after 3 consecutive months of follow-ups. The patients’ radiation results were assessed by X-ray (Figs. 4 and 5), while the knee joint and hip function were graded using the Harris hip score (HHS), Lysholm knee function score, and the visual simulation scale.

### Clinical data

The clinical data of patients before, during, and after surgery were collected and compared. Data collected before the operation included: the patient’s basic condition, injury mechanism, gender, BMI date, ASA, and fractal of fracture. During the Operation, data collected included the anesthesia type, operation time, blood loss, blood transfusion in surgery, and intraoperative fluoroscopy times. Post-surgery data collected included the time of surgery/hospital discharge, the occurrence of deep blood and wound infection, and the LKFS and VAS scores.

### Statistical analysis

The statistical analysis of this study was carried out using SPSS 23.0. Descriptive statistics of categorical variables included frequencies and percentages, while continuous variables were presented as mean ± SD/ mean ± SD (median). For continuous variables with a normal distribution, STUDENTS’ T-test was used to compare the two groups. When the data did not show a normal distribution, the Mann-Whitney U test was used. For categorical variables, Pearson’s chi-square test and Fisher’s exact chi-square test were used. Statistical significance was determined at P < 0.05.

## Results

Ordinary circumstances: A comparison of demographic and fracture characteristics between the two groups is presented in Table 1. A total of 44 eligible patients were enrolled from May 2018 to October 2022, 23 in the DRTR group and 21 in the traction table group. In the DRTR group, there were 16 AO/OTA patients with type A and 7 with type B, with an average age of 39.6 years. 17 patients had high-energy injuries and 6 had low-energy injuries, and the average follow-up time was 11.6 months. In the traction table group, there were 12 AO/OTA patients with type A and 9 patients with type B. The average age of patients was 37.6 years old, with 15 patients having high-energy injuries and 6 patients having low-energy injuries. The average follow-up time was 15.5 months. There were no significant differences in age, sex, fracture type, and injury mechanism between the two groups.

**Table 1:** Basic information on patients, Mechanism of injury, Gender, BMI, ASA, and Classification of fracture

	Total patient (n = 44)	DRTR (n = 23)	Traction table (n = 21)	P value
Gender (%)				0.870^a^
Male	34 (77.3)	18 (78.3)	16 (77.2)	
Female	10 (22.7)	5 (21.7)	5 (23.8)	
Age (years), mean ± SD	37.6 ± 17.8	39.6 ± 17.2	35.4 ± 18.6	0.192^c^
BMI, mean ± SD	23.2 ± 3.8	23.3 ± 3.8	23.1 ± 3.9	0.549^c^
ASA(%)				1.000^b^
1–2	37 (84.1)	19 (82.6)	18 (85.7)	
3–4	7 (15.9)	4 (17.4)	3 (14.3)	
Damage mechanism (%)				0.853^a^
High energy	32 (72.7)	17 (73.9)	15 (71.4)	
Low energy	12 (27.3)	6 (26.1)	6 (28.6)	
AO Classification (%)				0.392^a^
A	28 (63.6)	16 (69.5)	12 (57.1)	
B	16 (36.4)	7 (30.5)	9 (42.9)	
Follow-up time, mean ± SD	13.5 ± 5.6	11.6 ± 3.6	15.5 ± 6.7	0.054^c^

(a) Pearson Chi-Square test; (b) Fisher exact test; (c) Mann-Whitney U test; (d) Student t test

Intraoperative Situation: The data comparison for the two groups is displayed in Table 2. The DRTR group had a shorter average device assembly time and operative time compared to the traction table group, although this result was not statistically significant. The number of intraoperative fluoroscopy and the opening rate was significantly lower in the DRTR group than in the traction table group (P < 0.05). However, there were no statistically significant differences between the two groups in terms of intraoperative blood loss and the rate of reduction.

**Table 2:** Anesthesia method, Operation time, Blood loss, Intraoperative blood Transfusion, and Intraoperative fluoroscopy times

	Total patient(n = 44)	DRTR (n = 23)	Traction table (n = 21)	P value
Anesthesia (%)				0.599^a^
General	31 (70.5)	17 (73.9)	14 (66.7)	
Spinal	13 (29.5)	6 (26.1)	7 (33.3)	
Set up time, mean ± SD	43.75 ± 13	40.65 ± 12.5	47.1 ± 13	0.09^c^
Procedure time, mean ± SD	208.1 ± 63.5	207.1 ± 44.6	209.0 ± 80.6	0.65^c^
Anesthesia time, mean ± SD	261.8 ± 58.8	254.0 ± 38.8	270.1 ± 74.9	0.564^c^
Blood loss, mean ± SD	311.3 ± 219.3	269.6 ± 184.4	357.1 ± 248.6	0.189^d^
Intraoperative fluoroscopy times, mean ± SD	18.9 ± 5.2	15.0 ± 3.0	23.1 ± 3.6	<0.001^d^
The quality of reduction (%)				0.544^a^
Anatomic	29 (65.9)	17 (73.9)	12 (57.1)	
Acceptable	9 (20.4)	4 (17.4)	5 (23.8)	
Poor	6 (13.7)	2 (8.7)	4 (19.1)	
Open reduction (%)	10 (22.7)	2 (8.7)	8 (38.0)	0.02^b^

(a) Pearson Chi-Square test; (b) Fisher exact test; (c) Mann-Whitney U test; (d) Student t test

Postoperative Follow-Up: The follow up data for the two groups is displayed in Table 3. During postoperative follow-up, the DRTR group had a shorter average length of hospitalization than the traction table group, but this difference was not statistically significant (P = 0.11). Among all participants, there was only one patients in the traction table group suffered from the superficial reduction incision infection. And we disinfected the wound and changed dressing every day, and treated it with the antibiotic cefazolin. two weeks after surgery, the incision was healed. In terms of the Harris Hip Score and Lyshol Lysholm knee function scores, the DRTR group had higher than those of the traction table group, with statistically significant results. The pain scores of the two groups were not statistically significant. During postoperative hospitalization, there were 2 cases of DVT, 2 cases of soft tissue injury, and 1 case of femoral lateral cutaneous nerve injury in the traction table group, however, there were no similar complications in the DRTR group.

**Table 3:** Time from surgery to discharge, Occurrence of deep vein thrombosis, Postoperative wound infection, HHS, LKFS, and VAS scores

	Total patient(n = 44)	DRTR (n = 23)	Traction table (n = 21)	P value
Post-operative hospital’s day(days), mean ± SD	18.8 ± 12.4	16.0 ± 11.4	22.0 ± 12.9	0.11^d^
DVT	2	0	2	
superficial wound infections	1	0	1	
Harris Hip Score, mean ± SD	89.5 ± 2.6	90.3 ± 2.5	88.6 ± 2.4	0.019
Lysholm knee function score, mean ± SD	79.8 ± 3.6	80.9 ± 3.9	78.6 ± 2.9	0.049
visual analog scale, mean ± SD	2.1 ± 0.7	1.9 ± 0.7	2.3 ± 0.6	0.089
Perineal soft tissue injury	2	0	2	
Lateral femoral cutaneous nerve injury	1	0	1	

(a) Pearson Chi-Square test; (b) Fisher exact test; (c) Mann-Whitney U test; (d) Student t test

## Discussion

In the treatment of femoral shaft fractures, the eccentric fixation operation of plate internal fixation has been gradually replaced by the central fixation operation of intramedullary nailing, which is now viewed as the gold standard for femoral shaft fractures [7–9]. During intramedullary nail placement, it is critical to achieve the anatomic reduction of the fracture mass and correction of rotation and angulation deformity, as failure to do so can result in malunion or dysfunction of the lower limb. Consequently, it is a great challenge for orthopedic surgeons to achieve high-quality closed reduction and thus strong fixation nowadays [10].

The muscles around the femur are rich, making it difficult to maintain the stability of the force line of the femoral shaft by manual pulling during the operation to achieve anatomic reduction. Consequently, reliance on equipment is critical. A traction table is a most commonly used equipment to assist intramedullary nail surgery [11]. The principle of this is to conduct the traction force to the knee, then to the femoral shaft through the traction boots fixed in the foot, and simultaneously, the perineal column versus the perineum to generate the opposite resistance to the foot traction boots to maintain the stability of the femoral shaft.

However, there are still drawbacks despite the orthopedic traction bed being widely used. Firstly, the force is trans-joint and will gradually weaken in the process of force transmission, making it necessary to increase the traction force for the operations that require huge traction to maintain the femoral shaft fracture. The prolonged traction of great force can lead to crush injury, ulcer, or ischemic injury of the calf, foot, and ankle [12]. The blocking effect of the perineal column can cause swelling, numbness of the skin in the scrotum and perineal area, pudendal nerve damage, and other complications. Additionally, complications such as pudendal nerves peroneus communis, nervus cutaneus femoris lateralis, and osteofascial compartment syndrome have been reported [13–16]. Furthermore, affected limbs are normally suspended when the orthopedic traction table is in continuous traction, making it difficult to reduce the fracture by lower limb traction alone when the broken end is displaced back and forth. In this case, open reduction is often needed.

To address these shortcomings, our hospital introduced a new type of closed fracture resetter known as the Double Reverse Traction Rack (DRTR), which has been proven to be highly efficient in the treatment of femoral fractures [17]. According to Zhang [6], the inventor, DRTR has advantages such as an excellent reset effect, low opening rate, and fewer complications. However, there are still very few comparative studies on the clinical efficacy of DRTR and traction table in infra-isthmal femoral shaft fractures. Therefore, this study aimed to compare the preoperative, intraoperative, and postoperative data of DRTR and orthopedic traction table in middle and lower femoral shaft fractures to clarify the clinical efficacy of the two.

The fundamental difference between these two is that DRTR is bone traction while the traction table is based on skin traction (Fig. 1). The DRTR is achieved by the reaction force between the upper and lower Schantz needles (one on the anterior superior iliac spine and the other on the femoral condyle) to obtain continuous traction at the broken end of the femoral shaft fracture. In this study, there were 2 soft tissue injuries and 1 nerve injury in the bone traction table group, but these complications were avoided when the DRTR was used due to the absence of the perineal column and ankle boot.

It has been reported that the assembly time of DRTR is longer than the traction Table  [18]. However, in this study, there was no significant difference between the two groups (P > 0.05), when assembly time was compared from the start of disinfection to the completion of device preparation. The total surgical time in the DRTR group was significantly shorter than the traction table group, mainly due to the decreased assembly time. This is important for older patients, as it not only reduces the time of anesthesia but also the time of intraoperative bleeding, which is critical for postoperative recovery.

In terms of intraoperative bleeding, despite two additional incisions, the DRTR group had approximately 87ml less than the traction table group, which may be related to the opening rate. Although the difference between the groups was small, it may be of more statistical significance in future prospective studies with large sample sizes.

The DRTR group was significantly less than the traction table group in terms of intraoperative fluoroscopy times. This could be attributed to the Kirschner steel needle with its auxiliary reduction which could avoid open reduction and significantly reduce the fluoroscopy times, thus minimizing the exposure of the surgeon to radiation. There was no significant difference in the excellence rate of reduction between the two groups (P > 0.05), but the opening rate of the DRTR group was significantly lower than that of the traction table group (P < 0.05), which was consistent with previous studies [19]. This decrease in the opening rate not only lowers the trauma to patients but also reduces the incidence of wound infection.

There was one case of postoperative superficial wound infection in the orthopedic traction table group during postoperative hospitalization, which may be attributed to an enlarged incision. No significant differences were found in postoperative pain scores between the two groups, however, the Harris hip and knee scores were significantly higher in the DRTR group than in the traction table group. This could be because, in the traction table group, trans-joint traction is caused by continuous traction on the collateral ligament and tensor fascia lata of the knee joint, whereas the DRTR group was bone traction, thus the ligament muscles around the knee joint were not affected.

## Limitations

There are some limitations about this work. Firstly, the sample size of this study is relatively small, which may lead to data bias. What’s more, due to the recent introduction of DRTR in our hospital, the postoperative follow-up time of patients is limited. Therefore, the further investigation should be performed, which include more participants. And longer follow-up times will be introduced in future studies.

## Conclusion

Femoral shaft fractures in adults can be treated with DRTR-assisted AN-IMN fixation. This technique is beneficial as it increases the stability of the long axis of the femoral shaft and reduces the operation time, making it easier to fix with AN-IMN. Additionally, it requires fewer surgical operators, making it ideal for the busy orthopedic trauma field.

## Declare

The research was conducted in the absence of any commercial or financial relationships that could be construed as a potential conflict of interest.

## Data Availability

The datasets and materials are available from corresponding authors on reasonable request.

## Abbreviations

DRTR: Double reverse traction repositor
AN-IMN: Antegrade intramedullary nailing
RE-IMN: Retrograde intramedullary nailing
CPM: Joint continuous passive motion instrument
HHS: Harris hip score
BMI: Body mass index
ASA: American association of anesthesiologists
LKFS: Lysholm knee function score
VAS: Visual analogue scale
DVT: Deep vein thrombosis
